# Sampling Bias in the Molecular Epidemiology of Tuberculosis

**DOI:** 10.3201/eid0804.000444

**Published:** 2002-04

**Authors:** Megan Murray

**Affiliations:** Harvard School of Public Health, Boston, Massachusetts, USA

**Keywords:** tuberculosis, epidemiology, DNA fingerprinting

## Abstract

Among the goals of the molecular epidemiology of infectious disease are to quantify the extent of ongoing transmission of infectious agents and to identify host- and strain-specific risk factors for disease spread. I demonstrate the potential bias in estimates of recent transmission and the impact of risk factors for clustering by using computer simulations to reconstruct populations of tuberculosis patients and sample from them. The bias consistently results in underestimating recent transmission and the impact of risk factors for recent transmission.

Molecular epidemiology makes use of the genetic diversity within strains of infectious organisms to track the transmission of these organisms in human populations. It is used extensively to differentiate reactivation tuberculosis (TB), which is due to a remote infection, from disease caused by recently transmitted organisms. This approach is based on the concept that epidemiologically related organisms share similar or identical genetic fingerprints, while unrelated organisms differ at some genetic loci. Isolates of *Mycobacterium tuberculosis* that occur in clusters sharing similar fingerprints are thought to be caused by recently transmitted infection; those with unique fingerprints are thought to result from distantly acquired infection. Since the extent of recent transmission of an infectious disease often directly reflects the success of control measures (*1*,*2*), accurately assessing this quantity is of considerable public health importance.

In addition to distinguishing primary TB from reactivation disease, these molecular techniques have been used to identify risk factors for recent transmission in population-based epidemiologic studies (*3*). The goals of these investigations have been both to quantify the extent of ongoing transmission of *M. tuberculosis* and to identify host- and strain-specific risk factors for disease spread. Typically, these researchers have studied a specific population at risk for the disease by enrolling a cohort of persons with incident clinical TB, assessing these patients’ individual risk factors, and fingerprinting the TB isolates obtained from them (*4*–*11*). TB cases are then categorized as either clustered or unique; a cluster is usually defined as two or more patients whose isolates share an identical or near-identical DNA fingerprint, while unique cases are those with unmatched patterns (*12*). Clustered cases are assumed to share fingerprints as a result of recent spread of the organism among those in the cluster, while cases with unique patterns are assumed to be TB resulting from reactivated latent infection. These studies usually report the proportion of cases that are clustered within the cohort and use this result to infer the relative proportions of clustered and unclustered cases in the community from which the cohort was drawn.

Two different methods have been used to estimate the proportion of clustered cases. The first method, usually referred to as the “n” method, uses the number of all cases that fall into clusters as the estimator of clustered cases. The “n minus one” method assumes that one case per cluster is a case of reactivation TB and thus removes one case per cluster from the counts of “clustered” cases. The “n minus one” approach gives a number of clustered cases that is always less than that calculated by the n approach. Covariates associated with clustered fingerprints are taken to be host-specific risk factors for recent transmission of *M. tuberculosis*. The identification of these risk factors may provide specific targets for interventions designed to interrupt disease transmission.

These population-based molecular studies are often based on random or convenience samples drawn from available clinical isolates of *M. tuberculosis*. Implicit in the “population-based” approach to molecular epidemiology is the assumption that the results of studies based on these samples are reliable estimates of the parameters of interest in the population from which the sample was drawn. The criteria by which an estimate is judged to be reliable require that it be precise and unbiased, or, in other words, free from both major random and systematic error (*13*). Small samples usually render parameter estimates imprecise, or more vulnerable to the effects of chance, but do not specifically cause them to be systematically biased. When the parameter in question is a measure of clustering, however, the correct classification of each clustered case depends on other cases that share identical fingerprints being included in the sample. If these cases are not included because the sample is too small, clustered cases will be misclassified as unique and the resulting proportion of clustered cases will be underestimated. This, in turn, results in underestimation of the extent of recent transmission and overestimation of the extent of reactivation TB, as well as biased estimation of the effects of risk factors for transmission.

The magnitude of the bias incurred by sampling strategies depends both on the sampling fraction and the frequency distribution of sizes of clusters in the population. A recent simulation study of the influence of sampling on estimates of recent TB transmission demonstrated that an increase in sampling fraction yields an increase in the proportion of isolates identified as clustered (*14*). These simulations further showed that underestimation of clustering is more marked in populations of isolates that include small clusters than those with in which large clusters predominate. For this study, I extended this approach by using analytic methods in addition to simulations to estimate the magnitude of the bias introduced by commonly used sampling strategies in assessing the relative proportion of clustered and unclustered cases and in estimating the relative effect of potential risk factors for recent transmission.

## Methods

 The purpose of this study is to investigate biases inherent in estimating measures of clustering and risk factors for clustering when common sampling strategies are used to collect the empirical data. Since the true distributions of cluster sizes cannot be directly observed if sampling is not complete, I used a Monte Carlo simulation model to generate a variety of hypothetical cluster distributions based on simple assumptions about TB transmission. These distributions represent a wide range of potential data structures reflecting heterogeneous transmission parameters, contact networks, and sociodemographic variables. Accordingly, my aim here is not to model TB transmission dynamics with precision but to generate a collection of heterogeneous cluster distributions that could be used to demonstrate the effects of sampling, given a variety of potential transmission settings.

Generally, the microsimulation model enumerates a population of discrete individuals, each of whom is characterized by a vector of variables that affect risk for TB infection, for clinical disease, and for transmitting infection once infected. Persons are assigned to a series of social and physical spaces such as households, neighborhoods, and multineighborhood communities. The model also specifies the stochastic processes by which latent disease reactivates, infection progresses to primary TB, immunity is conferred by vaccination or by previous infection, and duration of disease is determined. Persons to whom disease is transmitted during the simulation acquire a variable reflecting the strain number of the source of their infection; thus, chains of disease transmission can be identified as “clusters” of cases sharing a specific strain number. The model is run over a time period during which these stochastic processes may occur. Output of the model includes standard measures of the incidence of infection and disease, the prevalence of infectious TB over time, and a count of cluster sizes. Five different cluster distributions were generated on the basis of running the model for 4 years with input variables specific to the different geographic and social settings in which TB is transmitted. The assumptions and baseline input variables for the model have been described (*15*).

### Estimation of Bias in Proportions of Unique and Clustered Cases

 The proportion of unique cases calculated after sampling and the variance of that proportion were estimated as follows. Using the “n” method to estimate the proportion of clustered cases, we assume that the true set of isolates is composed of *n_k_* clusters of size *k* for *k* = 1,2,…,*k*_max_. Further, we assume that each subject in the true set of isolates is sampled independently with a common sampling probability *p*.

Let I*_ijk_* be the indicator of whether the *i*^th^ subject *i* =1,...,*k* from the cluster, *j*=1,…, *n_k_*_,_ of size *k* has been sampled. Under our assumptions, the I*_ijk_* are i.i.d. *Bernoulli* (*p*) random variables. The total number of subjects sampled is *N*=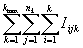
*.* Therefore, the expected value of the number of isolates is



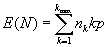

*.*


The variance of *N* is *var(N) = 
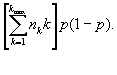
*

 Now let *U_jk_ =* 1 if the number of isolates sampled from the *j*^th^ cluster of size *k* is precisely 1 and U*_jk_* = 0 if otherwise. Then the total number of unique isolates is *U = 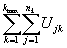
*. Now *U_jk_* is a Bernoulli random variable with success probability *kp(1-p)^k-1^* equal to the probability of choosing exactly one member from the *j*^th^ cluster of size k. Hence,



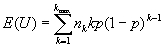



*var(U) = 
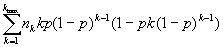
*

The expectation and large sample variance of the random variable (*U*/*N*) are derived in Appendix 1. Using these formulae for *E*(*U*/*N*) and *var*(*U*/*N*), estimates of the biased results for a range of sampling fractions were calculated for each of the five transmission scenarios described above. The results of this analysis were verified against a computer simulation that counted cluster sizes after random draws without replacing a proportion p of the true populations of isolates. For each true data set, the simulated data collection process was repeated 1,000 times. The mean value of the estimates obtained is reported, in addition to simulated confidence intervals expressed as values that represented the 0.05th and 0.95th largest estimates. The variance of the empirical distribution for the set of 1,000 simulations was nearly identical to that obtained by the large sample variance formula for a ratio expressed above.

 These simulations were repeated using the “n minus one” approach, in which one case per cluster is removed from the count of clustered cases and added to the count of reactivation cases. The analytic solution follows the same logic (Appendix 2).

### Estimation of Bias in the Relative Risks and Odds Ratios of Risk Factors for Recent Transmission

The magnitude of bias in the odds ratios of potential risk factors introduced by the misclassification of clustering due to sampling error was also assessed. Risk factors for clustering were postulated to which were assigned “true” odds ratios of 2, 5, and 10. The prevalence of these risk factors in the absence of clustering was set at 0.1. This exposure was thus randomly assigned to 10% of the unclustered cases and proportions of the clustered cases to obtain the specified odds ratios in each of the modeled data sets. The odds ratios were recalculated after sampling by moving the clustered cases that were sampled as unique from the category of recently transmitted cases to the category of reactivated cases and reassessing the respective exposure status for these outcomes.

## Results

### Bias in Estimates of Proportion of Unique and Clustered Cases

 Output from the transmission model (Table 1) includes estimates of the incidence of TB infection and clinical TB disease, as well as a summary of the frequency distribution of cluster size for each scenario. The proportion of unique isolates that would be observed after sampling a given fraction of the isolates in the complete data sets for each of the five scenarios was estimated analytically and verified by computer simulation. These methods produced nearly identical results, demonstrating that there is often substantial bias in the estimated proportions of unique and clustered cases of TB when sampling is based on sampling fractions consistent with those used in common epidemiologic practice. Table 2 summarizes the error in the measurement of the proportion of unique and clustered cases of TB introduced by sampling various fractions of the data from the range of transmission scenarios. These results are given for both the “n” and “n minus one” methods of counting clustered cases; they show that the error in these estimates depends on both the “true” transmission pattern and the fraction of the total data sampled. Transmission scenarios in which there is a higher “true” proportion of unique cases and those in which transmission is concentrated in large clusters tend to demonstrate less error than those in which there are fewer unique cases and more small clusters. In all cases in which the estimate is biased, the estimated proportion of unique cases is an overestimate of the true value, indicating that the error in these estimates tends to inflate the proportion of TB cases due to reactivation and minimize the proportion due to recent transmission. In many simulations, all of the 1,000 estimates obtained were less than the true proportion.

**Table 1:** Model-based output statistics from a microsimulation of tuberculosis transmission

Output statistics	High burden		Moderate burden		Low burden
Sudan	NY prison	Algeria	US prison	Netherlands
Tuberculosis incidence^a^	190	581	32	82	14
Consensus incidence estimates	200	NA^b^	44	NA^b^	10
ARI^c^	0.025	0.046	0.003	0.005	0.001
Maximum cluster size	87	19	9	17	15
Mean cluster size	10.2	3.2	1.7	2.9	1.7
Proportion of unique isolates	0.181	0.253	0.432	0.289	0.490

^a^Incidence per 100,000. Consensus incidence estimates are shown for comparison with estimates obtained from the model. ^b^No data available. ^c^ARI = Annual risk of infection.

**Table 2:** Monte Carlo means and 95% ranges for the proportion of unique isolates and for odds ratios after sampling a fraction of the complete data set.

Sampling fraction	1	0.7	0.5	0.1
**Sudan**				
Proportion of reactivated isolates				
n method	0.18	0.19 (0.18-0.19)	0.21 (0.19-0.23)	0.37 (0.30-0.43)
“n minus one” method	0.28	0.32 (0.30-0.34)	0.35 (0.30-0.41)	0.54 (0.47-0.62)
Odds ratios^a^	2	1.88 (1.87-1.97)	1.77 (1.66-1.88)	1.34 (1.28-1.45)
	5	4.18 (4.13-4.78)	3.51 (3.01-4.18)	1.84 (1.60-2.18)
	10	7.52 (7.38-9.27)	1.84 (1.67-1.84)	2.37 (2.08-2.99)
**New York prisons**				
Proportion of reactivated isolates				
n method	0.12	0.14 (0.12-0.16)	0.16 (0.13-0.20)	0.45 (0.28-0.62)
“n minus one” method	0.33	0.36 (0.33-0.38)	0.39 (0.32-0.45)	0.67 (0.61-0.73)
Odds ratios	2	1.77 (1.60-1.95)	1.62 (1.45-1.83)	1.16 (1.12-1.29)
	5	3.51 (2.73-4.58)	2.78 (2.18-3.83)	1.37 (1.25-1.37)
	10	5.79 (4.07-8.66)	4.17 (2.99-6.58)	1.58 (1.39-2.12)
**Algeria**				
Proportion of reactivated isolates				
n method	0.43	0.48 (0.45-0.51)	0.16 (0.13-0.20)	0.45 (0.28-0.62)
“n minus one” method	0.65	0.71 (0.69-0.74)	0.76 (0.69-0.83)	0.92 (0.81-0.99)
Odds ratios	2	1.81 (1.75-1.92)	1.67(1.45-1.97)	1.29 (1.18-1.73)
	5	3.68 (2.79-4.82)	3.02 (2.99-4.73)	1.98 (1.77-2.33)
	10	6.58 (5.55-8.23)	5.05 (4.17-6.58)	2.62 (2.26-3.28)
**U.S. prisons**				
Proportion of reactivated isolates				
n method	0.29	0.33 (0.29-0.39)	0.37 (0.29-0.48)	0.68 (0.35-1.00)
“n minus one” method	0.33	0.35 (0.31-0.38)	0.37 (0.32-0.41)	0.62 (0.50-0.73)
Odds ratios	2	1.8 (1.62-1.98)	1.67 (1.45-1.97)	1.29 (1.18-1.73)
	5	3.68 (2.79-4.82)	3.02 (2.99-4.73)	2.11 (1.62-5.31)
	10	6.86 (5.30-9.66))	4.67 (3.02-9.13)	2.11 (1.62-5.31)
**Netherlands**				
Proportion of reactivated isolates				
n method	0.49	0.62 (0.55-0.69)	0.62 (0.55-0.69)	0.89 (0.77-1.00)
“n minus one” method	0.65	0.78 (0.72-0.85)	0.78 (0.72-0.85)	0.93 (0.79-1.00)
Odds ratios	2	1.8 (1.62-1.98)	1.67 (1.57-1.81)	1.40 (1.31-1.49)
	5	3.68 (2.79-4.82)	3.05 (2.63-3.78)	2.02 (1.79-2.32)
	10	6.86 (5.30-9.66))	4.76 (3.86-6.47)	3.86 (2.39-3.25)

^a^Confidence intervals for odds ratios are based on the results of 2 by 2 tables, with data adjusted for the mean misclassification introduced by sampling.

### Bias in Odds Ratios for Risk Factors for Clustering

 The bias in the proportions of clustered and unclustered cases results from misclassification of cluster status due to inadequate sampling; this misclassification also biases the results of analyses of risk factors for recent transmission in the direction of the null hypothesis of no effect. Table 2 also presents estimates of the odds ratios for the effect of a range of hypothetical risk factors for recent transmission. These results show that the odds ratios of a risk factor for clustering are markedly underestimated in the transmission scenarios in which there are lower proportions of unique cases or in which smaller cluster predominate. This bias is especially marked when odds ratios are high; in the worst-case scenario described in Table 2, an odds ratio of 10 could be estimated as 1.58 when only 10% of the isolates are sampled.

## Discussion

 The recent development of molecular methods to accurately type infectious organisms has led to a marked proliferation in studies of the molecular epidemiology of infectious diseases, especially of TB. The goals of many of these studies have been to address the longstanding problem of assessing the relative proportions of incident TB cases due to recent transmission and to chronic or reactivated disease and to identify risk factors for recent transmission. A systematic bias that consistently underestimates the proportion of cases due to recent transmission could present a serious impediment to the constructive use of molecular typing techniques for studying the epidemiology of infectious disease.

 The results of this study show the extent to which bias can be introduced by sampling strategies commonly used in the molecular epidemiology of TB. Depending on the underlying distribution of cluster sizes, the error involved in underestimating the proportion of unique TB isolates in a sample may be sizable, even when up to 70% of the complete data is sampled. The odds ratios for risk factors for clustering are also consistently and markedly underestimated with this approach. The findings of this study support the conclusions of previous investigators (*14*) who have shown that the extent of error in these estimates is a function of both sampling fraction and underlying cluster distribution in the complete data sets. These results imply that reasonable predictions of the extent of error can be made, given knowledge of both the true distribution of cluster sizes in the population of persons with TB and the size of the population of TB patients from which the sample was drawn. Although the true distribution of cluster sizes cannot be observed in the absence of complete sampling, epidemic models such as this one may elucidate factors that contribute to these distributions and help investigators arrive at prior expectations of cluster distributions in the specific transmission scenarios under study.

I considered how much impact this kind of sampling bias might have had on the studies of the molecular epidemiology of TB published to date. Many researchers report on a convenience sample of cases drawn from one or more clinical sites, without providing an estimate of the number of incident cases in the area in question during the period in which the cases were collected (*16*–*19*). In areas with high TB prevalence, the number of cases in these series is often <1% of the number of cases expected in that region on the basis of national reporting or World Health Organization predictions. These results suggest that the bias expected in these studies is so extreme that the findings are useful only as lower bounds for the proportion of recently transmitted cases and for risk factors for recent transmission. Nonetheless, lower bounds may be informative in situations in which undetected transmission is incorrectly attributed to reactivation disease alone or when a new risk factor for transmission is identified.

In industrialized countries with lower rates of incident TB, researchers have tried to enroll a compete cohort of patients by making use of public health reporting systems to identify and fingerprint all new cases of clinical TB in a defined geographic region during a specified time period (*4*,*5*,*20*–*22*). Although this approach leads to much more complete and systematic sampling, it may not always ensure that the resulting estimates are free from bias. For these series of cases to be complete samples, one would have to assume that none of the cases in the sample had transmission links to cases that did not appear in the study population or were reported before the onset of the study. Furthermore, the most rigorously documented TB fingerprinting studies have reported 15%-40% loss of data as a result of difficulties in culturing, fingerprinting, and interpreting fingerprint patterns (*4*,*5*,*20*–*22*). Even if the patients excluded from these studies resemble those retained in every other respect, their exclusion will result in a biased outcome of the study.

 The “complete” data sets used to estimate bias in this study were generated through stochastic epidemic modeling that outputs cluster distributions in addition to estimates of the incidence of TB infection and disease. Multiple demographic and disease-specific parameters have been found to affect cluster distributions, and many potential “transmission scenarios” could be generated by varying these parameters. In addition, the length of the study period and the stability of the molecular markers used will impact the observed patterns of clustering (*23*,*24*). Given that true cluster distributions cannot be known in the absence of complete sampling, the model cannot be validated by using it to derive known cluster distributions. Since the purpose of this study is to explore the bias in measures commonly used in empirical studies of molecular epidemiology, sets of parameters were chosen from a variety of specific areas in which the burden of TB disease has been described or projected based on the information currently available. Although the true transmission patterns in any particular population may be inadequately captured by the epidemic model used, these results do provide some perspective on the potential misinterpretation of molecular data on TB. The simulations may also differ from data sets obtained in the field in that sampling was random and the very real problem of selection bias in the collection of isolates was not addressed. Finally, in the assessment of the bias in the estimates of the effect of risk factors for clustering, I assigned risk status randomly within groups of clustered and unique cases. If cluster size were correlated with a risk factor for clustering, so that, for example, incarceration was more common among cases in large clusters than small ones, the bias in the odds ratio of incarceration would be less than the estimates reported here.

 These results demonstrate that estimates of clustering based on molecular fingerprinting of a population of isolates of infectious agents may be severely biased. When these methods are used to estimate the extent of primary and reactivation disease in a community, they consistently underestimate recent transmission. In circumstances in which the error is greatest, the bias may undermine the value of an investigation by providing a community with false reassurance that ongoing transmission is being curtailed and therefore that control measures are adequate.

The findings of this study further suggest that molecular methods in epidemiology require the development of both appropriate epidemiologic study design and analytic tools to yield meaningful assessments of disease transmission. In particular, they imply that estimates of recent transmission obtained by molecular methods cannot be compared across studies which have used different sampling fractions and in which the distribution of cluster size can reasonably be expected to vary. One way for molecular epidemiologists to approach this problem is to provide sensitivity analyses estimating the potential error involved, given prior expectations of cluster distributions and an estimate of the fraction of cases sampled in a particular study. The analytic solution presented here can be easily programmed and used to explore the range of potential error under a variety of hypothetical transmission scenarios.

## Appendix 1

 We wish to derive the expectation and variance of the random variable *U/N,* denoting the proportion of all sampled isolates that form a unique cluster of size 1 in the sample. In large samples, the mean of *U/N* is approximately the ratio of the mean of *U* to the mean of *N*

E
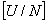


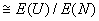


 and the variance of *U/N* is approximated by the large sample variance formula for a ratio.

*var*(*U/N) 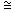
*

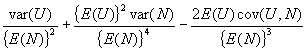


It only remains to evaluate *cov*(*U,N*), which is done in the following lemma.

Lemma: Under our assumptions,


*cov(U,N) = 
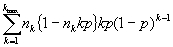
*


Proof: by independence, *cov(U,N) = 
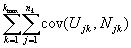
* where 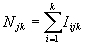
*.* Now, *cov*(*U_jk_,N_jk_*) *= E*(*U_jk_, N_jk_*) *– E*(*U_jk_*)*-E*(*N_jk_*) *= E*(*U_jk_*)*- E*(*U_jk_*)*-E*(*N_jk_*)*= E*(*U_jk_*)[1- *E*(*N_jk_*)] where *E*(*U_jk_*) = *kp*(1-*p*)*^k^* ^– 1^ and *E*(*N_jk_*) = *n_k_p.* The result then follows.

## Appendix 2

The bias in the proportion of reactivated cases after sampling when the clustered cases are counted by using the “n minus one” method is described below. The number of cases considered to be due to reactivation is the sum of the unique cases and the source cases. The “true” number of source cases is equal to the number of clusters in the complete data set, 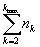.

We are interested in finding the number of source cases after sampling. Since the number of source cases in a sample is equal to the “true” number of source cases minus the source cases that are not sampled or are sampled as unique, we need to estimate the expected value of the numbers of clusters not sampled and the expected value of the clusters sampled as unique. Let E(CL0) and E(CL1) be the expected values of the numbers of clustered not sampled or sampled as unique, respectively. Then, by using the nomenclature defined in the text and following the logic there described:

E(CL0) = 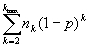 and E(CL1) = 
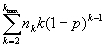


The expected number of source cases after sampling a fraction *p* of the complete set of isolates is equal to


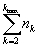
 - 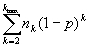 - 
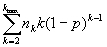


The overall estimate of the proportion of reactivated cases can then be obtained by summing the number of unique cases after sampling with the number of source cases and dividing by the expected number of sampled isolates, *p*(N).

## References

[1] Rieder HL. Methodological issues in the estimation of the tuberculosis problem from tuberculin surveys. Tuber Lung Dis. 1995;76:114–21. 10.1016/0962-8479(95)90552-9 7780092

[2] Chin DP, Crane CM, Diul MY, Sun SJ, Agraz R, Taylor S, Spread of *Mycobacterium tuberculosis* in a community implementing recommended elements of tuberculosis control. JAMA. 2000;283:2968–74. 10.1001/jama.283.22.2968 10865275

[3] Small PM, Moss A. Molecular epidemiology and the new tuberculosis. Infect Agents Dis. 1993;2:132–8. 7909707

[4] Alland D, Kalkut GE, Moss AR, McAdam RA, Hahn JA, Bosworth W, Transmission of tuberculosis in New York City; a population based study using conventional and molecular methods. N Engl J Med. 1994;330:1710–6. 10.1056/NEJM199406163302403 7993412

[5] Small PM, Hopewell PC, Singh SP, Paz A, Parsonnet J, Ruston DC, The epidemiology of tuberculosis in San Francisco analysis by DNA fingerprinting and conventional epidemiologic methods. N Engl J Med. 1994;330:1703–9. 10.1056/NEJM199406163302402 7910661

[6] Wilkinson D, Pillay M, Crump J, Lombard C, Davies GR, Sturm AW. Molecular epidemiology and transmission dynamics of *Mycobacterium tuberculosis* in rural Africa. Trop Med Int Health. 1997;2:747–53. 10.1046/j.1365-3156.1997.d01-386.x 9294544

[7] Hermans PWM, Messadi F, Guebrexabher H, van Soolingen D, de Haas PE, Heersma H, Analysis of the population structure of mycobacterium tuberculosis in Ethiopia, Tunisia, and the Netherlands: Usefulness of DNA typing for global tuberculosis epidemiology. J Infect Dis. 1995;171:1504–13. 7769285 10.1093/infdis/171.6.1504

[8] Chevral-Dellagi D, Abderrahman A, Haltiti R, Koubaji H, Gicquel B, Dellagi K. Large-scale DNA fingerprinting of *Mycobacterium tuberculosis* strains as a tool for epidemiological studies of tuberculosis. J Clin Microbiol. 1993;31:2446–50. 8104956 10.1128/jcm.31.9.2446-2450.1993 PMC265776

[9] Yang ZH, de Haas PEW, Wachmann C, van Soolingen D, van Embden JDA, Anderson AB. Molecular epidemiology of tuberculosis in Denmark in 1992. J Clin Microbiol. 1995;33:2077–81. 7559951 10.1128/jcm.33.8.2077-2081.1995 PMC228338

[10] van Deutekon H, Gerritsen JJ, van Soolingen D, van Ameijden EJC, van Embden JDA, Coutinho RA. A molecular epidemiologic approach to studying the transmission of tuberculosis in Amsterdam. Clin Infect Dis. 1997;25:1071–7. 10.1086/516072 9402360

[11] Chaves F, Dronda F, Cave MD, Alonso-Sanz M, Gonzalez-Lopez A, Eisenach KD, A longitudinal study of transmission of tuberculosis in a large prison population. Am J Respir Crit Care Med. 1997;155:719–25. 9032218 10.1164/ajrccm.155.2.9032218

[12] Small P, Behr M. Molecular fingerprinting of *Mycobacterium tuberculosis*: how can it help the clinician? Clin Infect Dis. 1997;25:806–10. 10.1086/515550 9356792

[13] Hogg RV, Tanis EA. Sampling distribution theory in probability and statistical inference. 5th edition. Upper Saddle River, NJ: Prentice-Hall, 1997: 237-82.

[14] Glynn JR, Vynnycky E, Fine PEM. Influence of sampling on estimates of clustering and recent transmission of *Mycobacterium tuberculosis* derived from DNA fingerprinting techniques. Am J Epidemiol. 1999;149:366–71. 10025480 10.1093/oxfordjournals.aje.a009822

[15] Murray MB. Determinants of cluster distribution in the molecular epidemiology of tuberculosis. Proc Natl Acad Sci U S A. 2002. In press. 10.1073/pnas.022618299 PMC122226 11818527

[16] Torrea G, Levee G, Grimont P, Martin C, Chanteau S, Gicquel B. Chromosomal DNA fingerprinting analysis using the insertion sequence IS6110 and the repetitive element DR as strain-specific markers for epidemiologic study of tuberculosis in French Polynesia. J Clin Microbiol. 1995;33:1899–904. 7665667 10.1128/jcm.33.7.1899-1904.1995 PMC228294

[17] Pineda-Garcia L, Ferrera A, Hoffner SE. DNA fingerprinting of *M. tuberculosis* strains from patients with pulmonary tuberculosis in Honduras. J Clin Microbiol. 1997;35:2393–7. 9276422 10.1128/jcm.35.9.2393-2397.1997 PMC229974

[18] Barnes PF, El-Hajj H, Preston-Martin S, Cave MD, Jones BE, Otaya M, . Transmission of tuberculosis among the urban homeless. JAMA. 1996;275:305–7. 10.1001/jama.275.4.305 8544271

[19] van Soolingen D, Qian S, de Haas PEW, Douglas JT, Traore H, Portaels F, Predominance of a single genotype of *Mycobacterium tuberculosis* in countries of East Asia. J Clin Microbiol. 1995;33:3234–8. 8586708 10.1128/jcm.33.12.3234-3238.1995 PMC228679

[20] Genewein A, Telenti A, Bernasconi C, Mordasini C, Weiss S, Maurer A, Molecular approach to identifying route of transmission of tuberculosis in the community. Lancet. 1993;342:841–4. 10.1016/0140-6736(93)92698-S 8104275

[21] Braden CR, Templeton GL, Cave MD, Valway S, Onorato IM, Castro KG. Interpretation of restriction fragment length polymorphism analysis of *Mycobacterium tuberculosis* isolates from a state with a large population. J Infect Dis. 1997;175:1446–52. 9180185 10.1086/516478

[22] Borgdorff MW, Nagelkerke N, van Soolingen D, de Haas PEW, Veen J, van Embden JDA. Analysis of tuberculosis transmission between nationalities in the Netherlands in the period 1993-1995 using DNA fingerprinting. Am J Epidemiol. 1998;147:187–95. 9457010 10.1093/oxfordjournals.aje.a009433

[23] Glynn JR, Bauer J, deBoer AS, Borgdorff MW, Fine PE, Godfrey-Faussett P, Interpreting DNA fingerprint clusters of *Mycobacterium tuberculosis*. European Concerted Action on Molecular Epidemiology and Control of Tuberculosis. Int J Tuberc Lung Dis. 1999;3:1055–60. 10599007

[24] Van Soolingen D, Borgdorff MW, de Haas PEW, Sebek MM, Veen J, Dessens M, Molecular epidemiology of tuberculosis in the Netherlands: a national study from 1993 to 1997. J Infect Dis. 1999;180:726–36. 10.1086/314930 10438361

